# Supplementary material for: Bacterial outer membrane vesicle based versatile nanosystem boosts the efferocytosis blockade triggered tumor-specific immunity
Source: Nat Commun. 2023 Mar 25;14:1675. doi: 10.1038/s41467-023-37369-0 (PMC10039929; doi:10.1038/s41467-023-37369-0)
Supplement: Supplementary file 1 — Supplementary Information [file 41467_2023_37369_MOESM1_ESM.pdf]

Supporting information

Bacterial Outer Membrane Vesicle Based Versatile Nanosystem Boosts the Efferocytosis Blockade Triggered Tumor-Specific Immunity

Wan-Ru Zhuang[1], Yunfeng Wang[1], Weidong Nie[1], Yao Lei[1], Chao Liang[1], Jiaqi He[1], Liping Zuo[1], Li-Li Huang[2], Hai-Yan Xie[1,*]

[1] School of Life Science, Beijing Institute of Technology, Beijing 100081, P.R. China

[2] School of Medical Technology, Beijing Institute of Technology, Beijing 100081, P.R. China

E-mail: hyanxie@bit.edu.cn

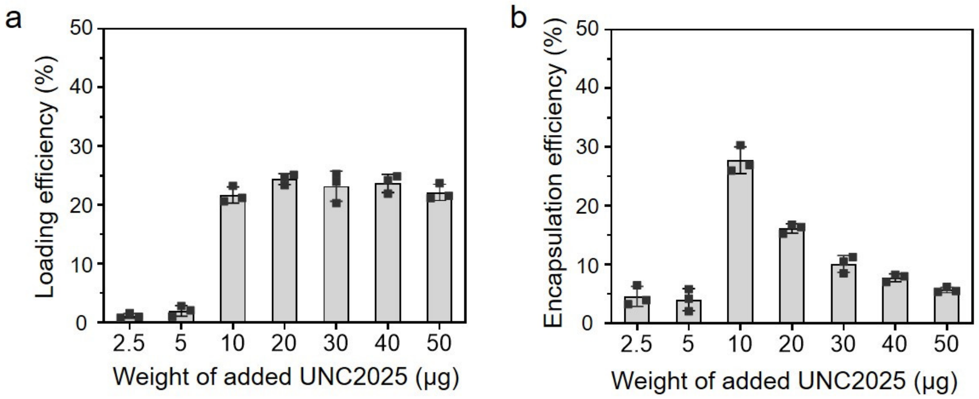

Supplementary Figure 1 Loading efficiency (**a**) and encapsulation efficiency (**b**) of UNC2025 in OMVs. Different amounts of UNC2025 were individually incubated with the same amount of OMVs (10 μg). The loading was saturated when 10 μg UNC2025 was added. Data are presented as mean ± s.d. (n = 3 biologically independent samples). Source data are provided as a Source Data file.

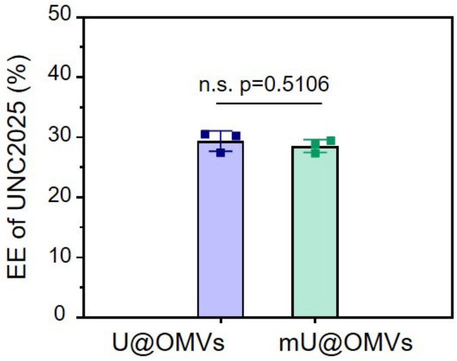

Supplementary Figure 2 Encapsulation efficiency (EE) of UNC2025 after functionalizing U@OMVs with Mal as quantified by UV-vis spectrum. Data are presented as mean ± s.d. (n = 3 biologically independent samples). Statistically significant differences between groups were identified by unpaired two-tailed Student's t-test. n.s., not significant. Source data are provided as a Source Data file.

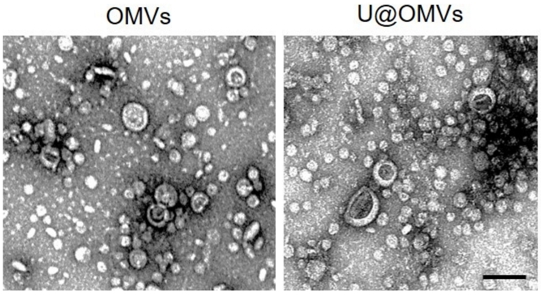

Supplementary Figure 3 TEM images of OMVs and U@OMVs. Scale bar: 100 nm. The experiments were repeated three times independently.

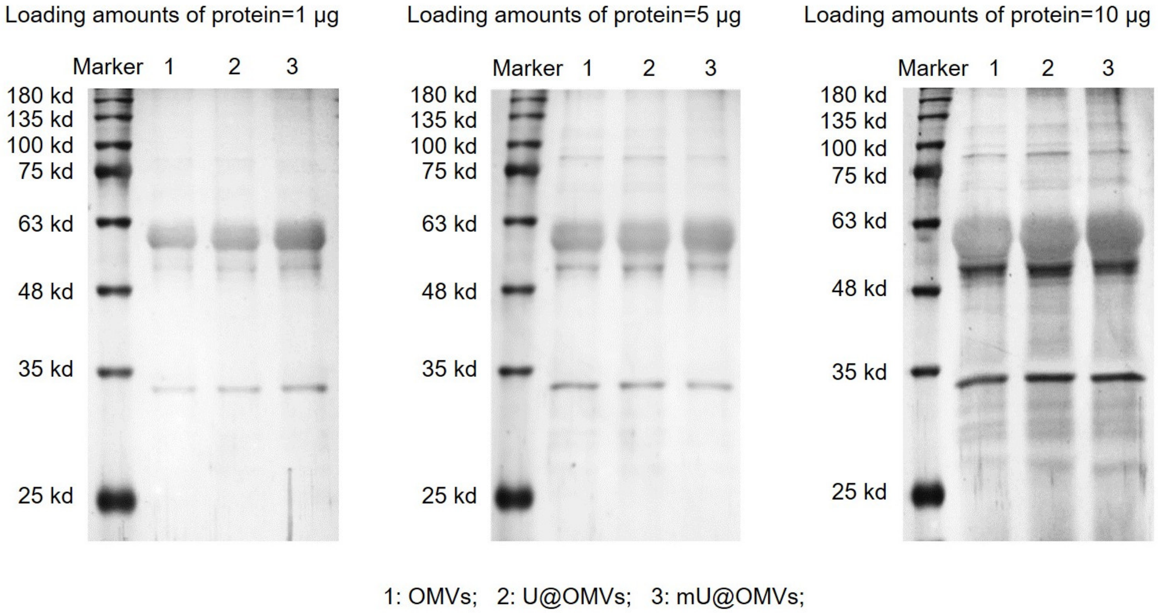

Supplementary Figure 4 Silver stain results of OMVs, U@OMVs and mU@OMVs with different amounts of proteins. The experiments were repeated three times independently.

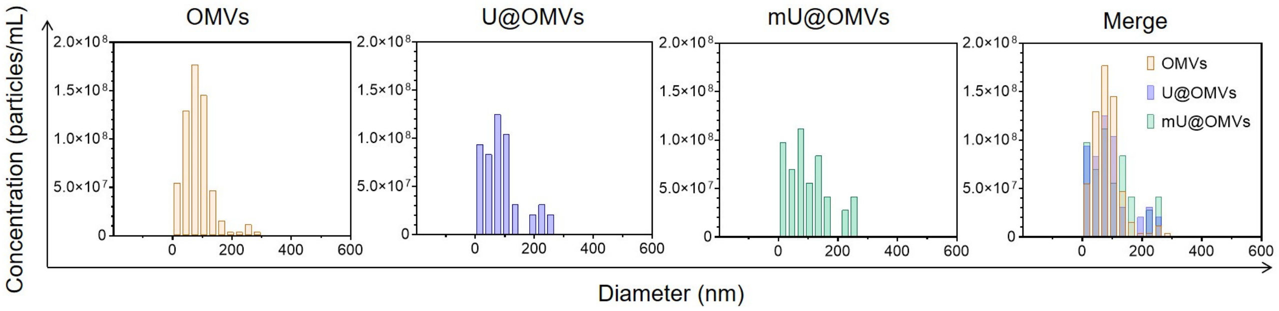

Supplementary Figure 5 Nanoparticle tracking analysis (NTA) results of OMVs, U@OMVs and mU@OMVs. Source data are provided as a Source Data file.

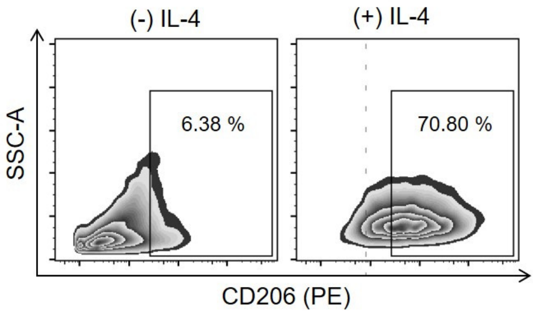

Supplementary Figure 6 Flow cytometry analysis of phenotype transformation of macrophages. 70.80 % of RAW264.7 cells were CD206 positive after the incubation with interleukin-4 (IL-4) for 48 h, indicating the efficient transformation of M0 to M2.

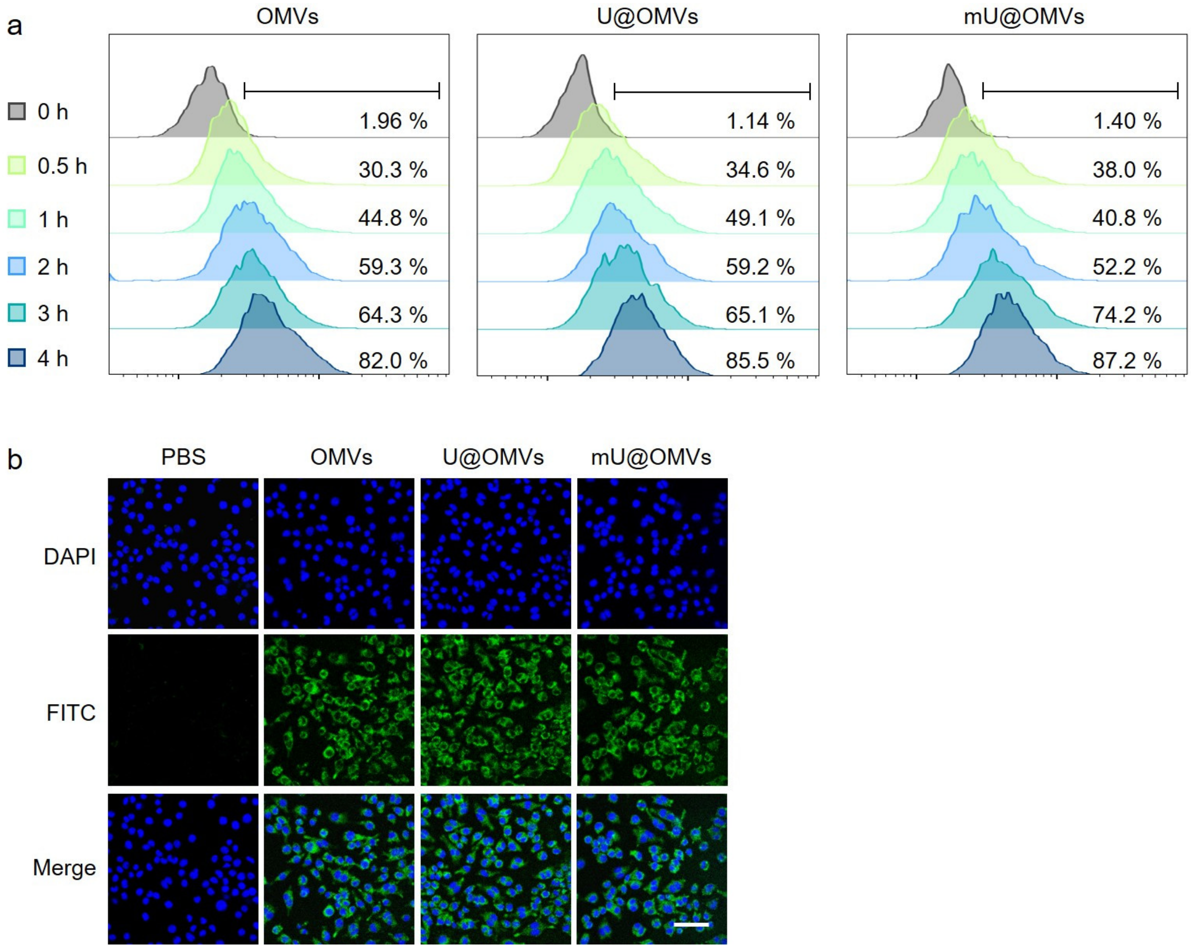

Supplementary Figure 7 Cellular uptake of OMVs, U@OMVs and mU@OMVs in M2 macrophages. **a** Representative flow cytometry plots of different vesicles at different time points. **b** Confocal image of cell uptake of different vesicles after incubation for 4 h. Vesicles (FITC-conjugated anti-E. coli LPS labeled, green); cell nucleus (Hoechst 33342, blue). Scale bar: 50 μm. The experiments were repeated three times independently.

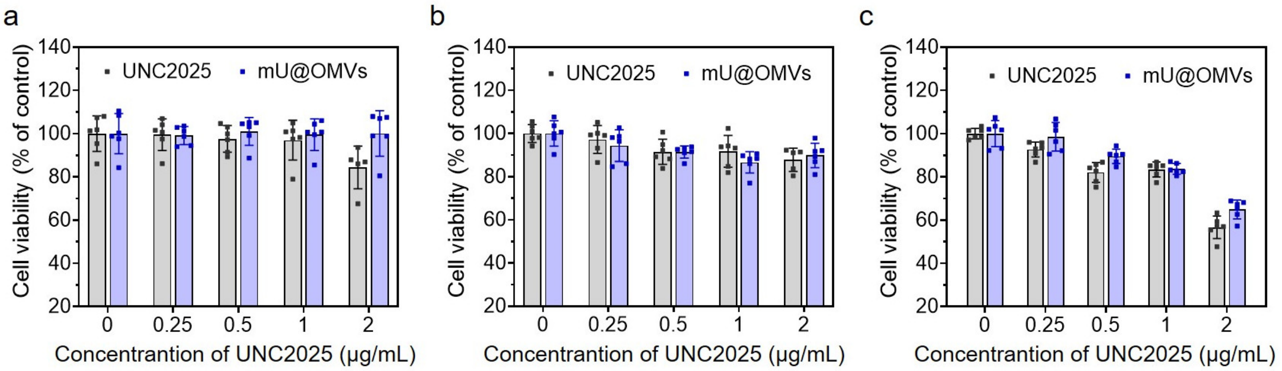

Supplementary Figure 8 Viabilities of RAW264.7 (**a**), B16F10 (**b**) and CT26 (**c**) cells after the incubation with different concentrations of UNC2025 and mU@OMVs for 24 h. Data are presented as mean ± s.d. (n = 6 biologically independent samples). Source data are provided as a Source Data file.

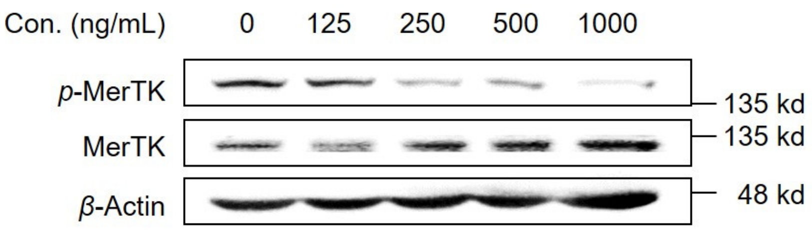

Supplementary Figure 9 Western blot analysis of the phosphorylation of MerTK in M2 macrophages after treatment with different concentrations of free UNC2025 for 2 h. The experiments were repeated three times independently.

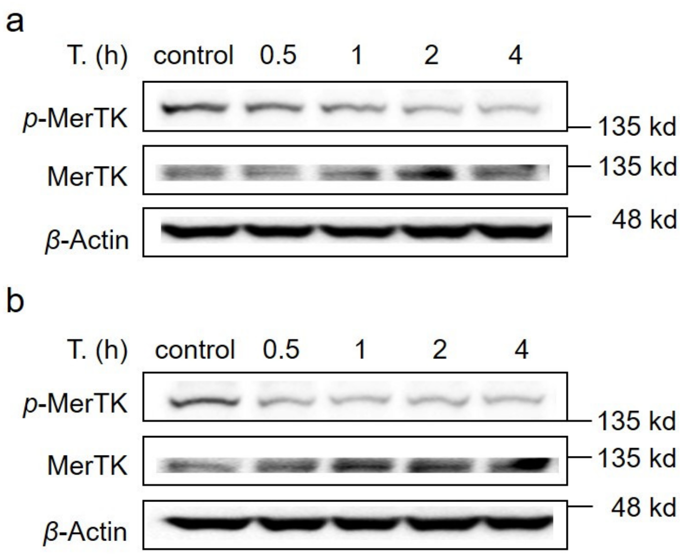

Supplementary Figure 10 Western blot analysis of the phosphorylation of MerTK in M2 macrophages after 1000 ng/mL free UNC2025 (**a**) or mU@OMVs (containing 1000 ng/mL UNC2025) (**b**) treatment for different incubation time. Source data are provided as a Source Data file. The experiments were repeated three times independently.

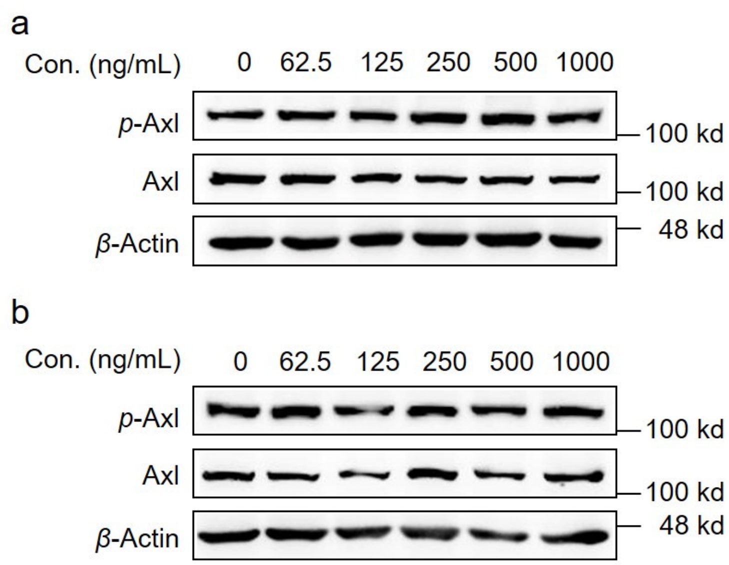

Supplementary Figure 11 Western blot analysis of the phosphorylation of Axl in M2 macrophages after treatment with different concentrations of free UNC2025 (**a**) or mU@OMVs (**b**) for 2 h. The result proved that small molecule inhibitor UNC2025 was specific for MerTK rather than Axl. Source data are provided as a Source Data file. The experiments were repeated three times independently.

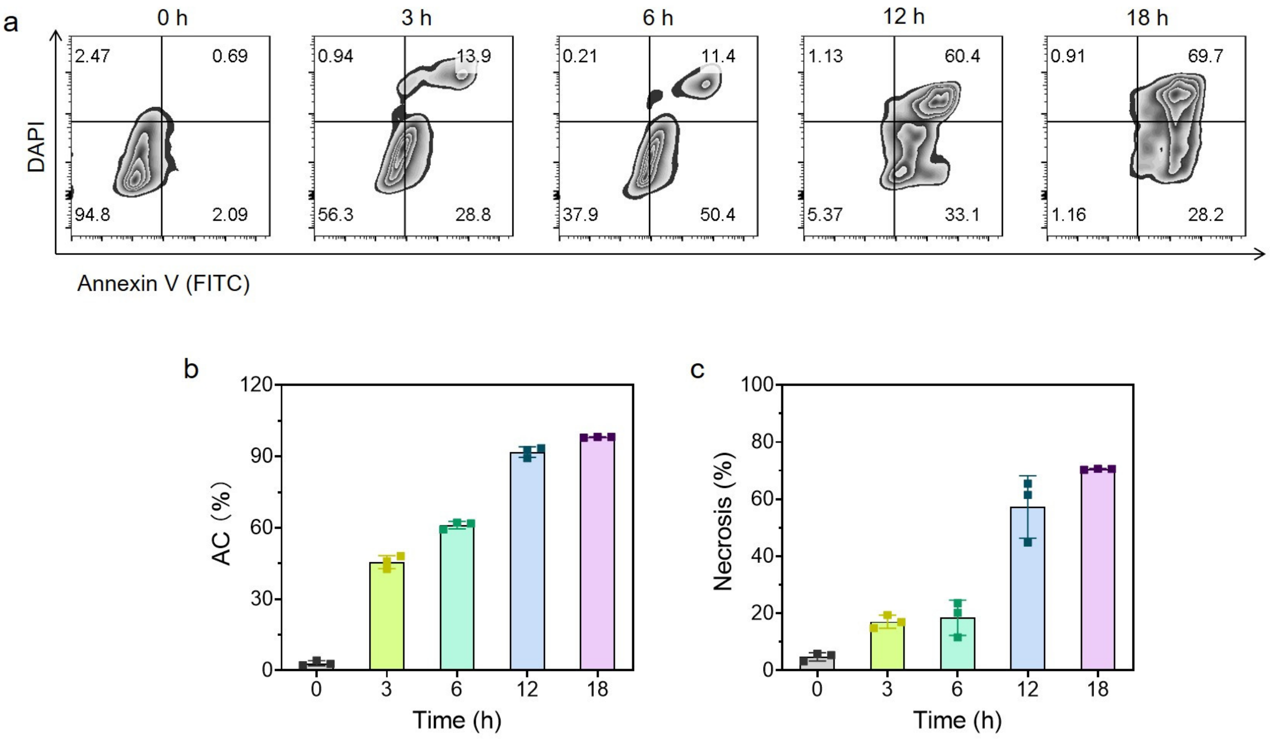

Supplementary Figure 12 Flow cytometry analysis of cell apoptosis by Annexin V-FITC/DAPI double staining in B16F10 melanoma cells treated with 5 μM doxorubicin for 0, 3, 6 h, 12 h, or 18 h. **a** Representative flow cytometry plots. **b** Quantification of apoptotic tumor cells (AC) at the indicated time points (Annexin V^+ tumor cells). **c** Quantification of necrotic tumor cells at the indicated time points ($DAPI^+$ tumor cells). Therefore, the final apoptotic tumor cells were obtained by adding 5 μM doxorubicin to B16F10 cells for 6 h, since around 60 % of B16F10 cells exposed PtdSer on the surface along with little necrosis (< 20 %) at this timepoint. Data are presented as mean ± s.d. (n = 3 biologically independent samples). Source data are provided as a Source Data file.

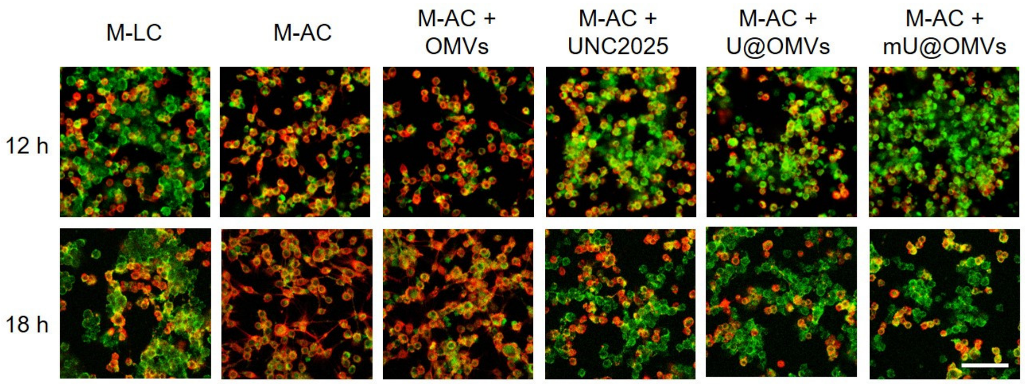

Supplementary Figure 13 CLMS imaging of the phagocytosis of AC (carboxyfluorescein succinimidyl ester (CFSE)-labeled, green) by macrophages (APC-conjugated anti-CD11b labeled, red) after a 12-hour or 18-hour incubation. M: M2 macrophages; LC: living B16F10 cells; AC: apoptotic B16F10 cells. Scale bar: 50 μm. The experiments were repeated three times independently.

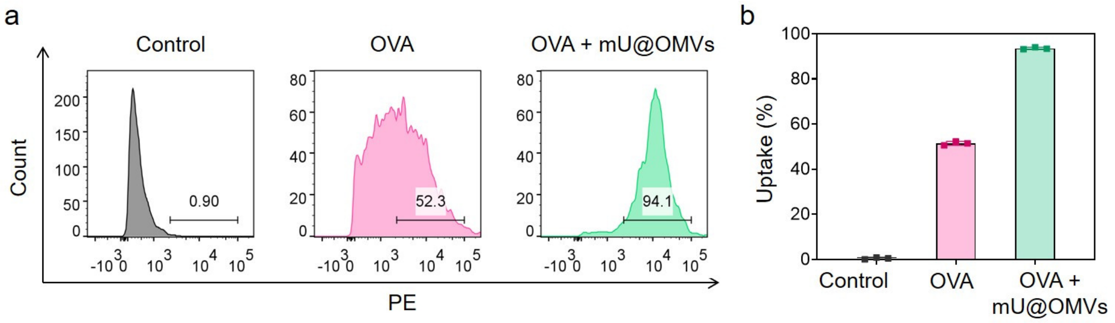

Supplementary Figure 14 Flow cytometry analysis (**a**) and quantification (**b**) of the internalization of OVA or OVA+mU@OMVs in BMDCs. Data are presented as mean ± s.d. (n = 3 biologically independent samples). Source data are provided as a Source Data file.

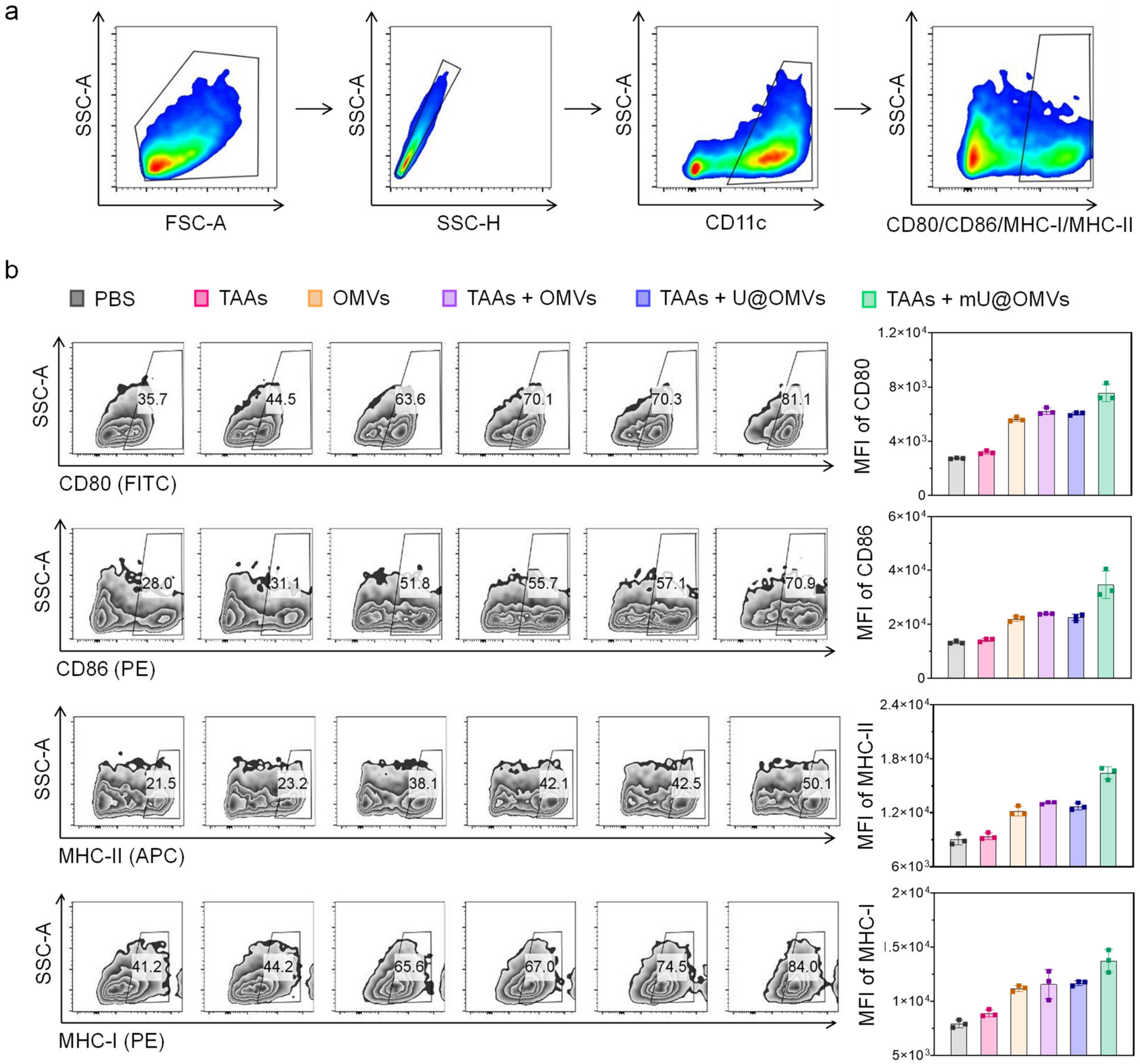

Supplementary Figure 15 a The gating strategy for flow cytometry analysis of CD11c⁺ CD80⁺/CD86⁺/MHC-II⁺/MHC-I⁺ DCs in BMDCs. **b** Representative flow cytometry plots of CD80, CD86, MHC-II or MHC-I positive cells (gated on CD11c⁺ cells) of BMDCs after different treatments. Data are presented as mean ± s.d. (n = 3 biologically independent samples). Source data are provided as a Source Data file.

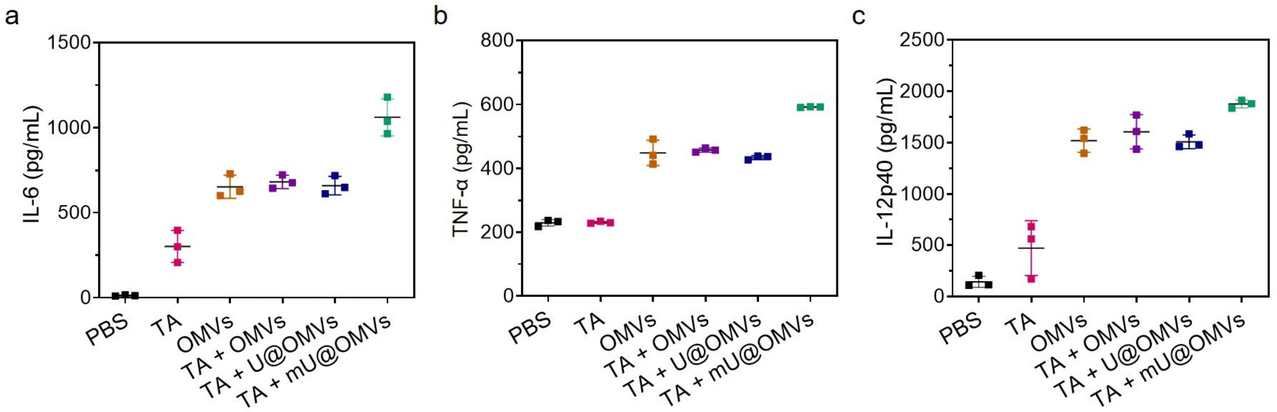

Supplementary Figure 16 Secretion of IL-6 (**a**), TNF-α (**b**), and IL-12p40 (**c**) by BMDCs determined by ELISA. Data are presented as mean ± s.d. (n = 3 biologically independent samples). Source data are provided as a Source Data file.

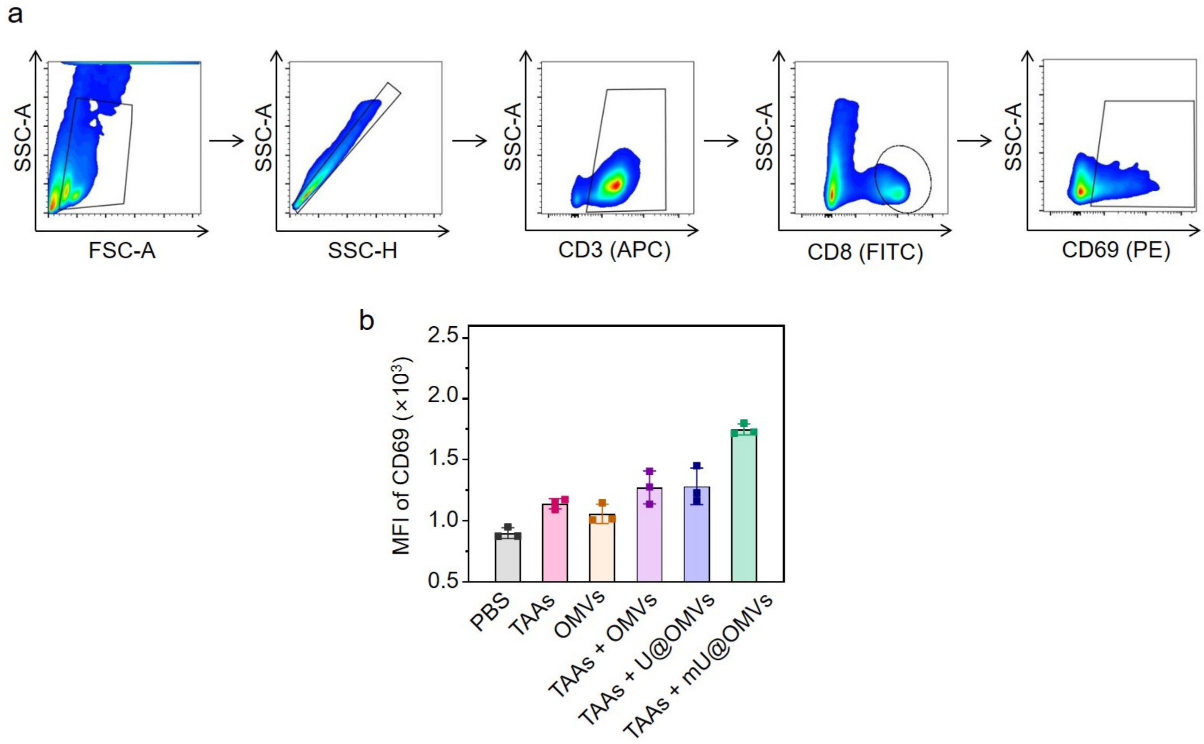

Supplementary Figure 17 Naive T cells are isolated from the spleens of C57BL/6 mice and co-cultured with BMDCs for 24 h. **a** The gating strategy for flow cytometry analysis of $CD3^+$ $CD8^+$ $CD69^+$ T cells. **b** MFI of CD69 (a costimulatory molecule for T cell activation) expressed on T cells were analyzed by flow cytometry (gated on $CD8^+$ cells). Data are presented as mean ± s.d. (n = 3 biologically independent samples). Source data are provided as a Source Data file.

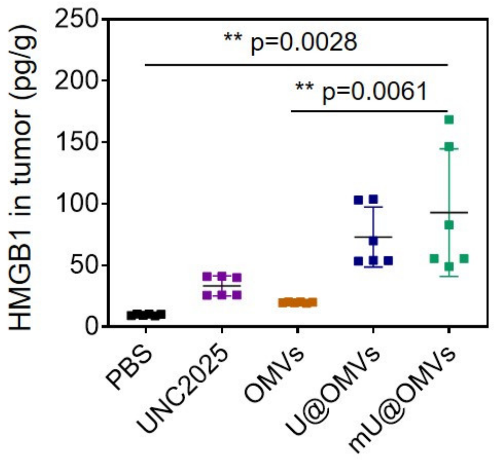

Supplementary Figure 18 HMGB1 levels in tumor tissues after different treatments determined by ELISA. Data are presented as mean ± s.d. (n = 6 biologically independent samples). Statistically significant differences between groups were identified by unpaired two-tailed Student's t-test. ****P < 0.0001, ***P < 0.001, **P < 0.01, *P < 0.05, n.s., not significant. Source data are provided as a Source Data file.

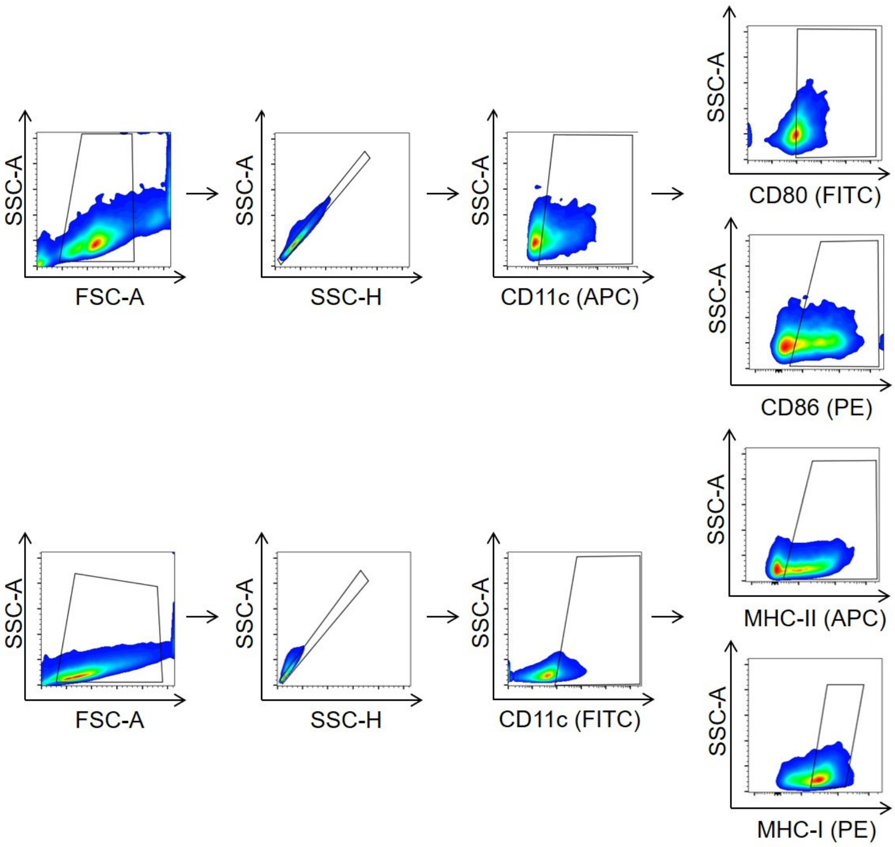

Supplementary Figure 19 The gating strategy for flow cytometry analysis of CD11c$^+$ CD80$^+$/CD86$^+$/MHC-II$^+$/MHC-I$^+$ DCs in DLNs.

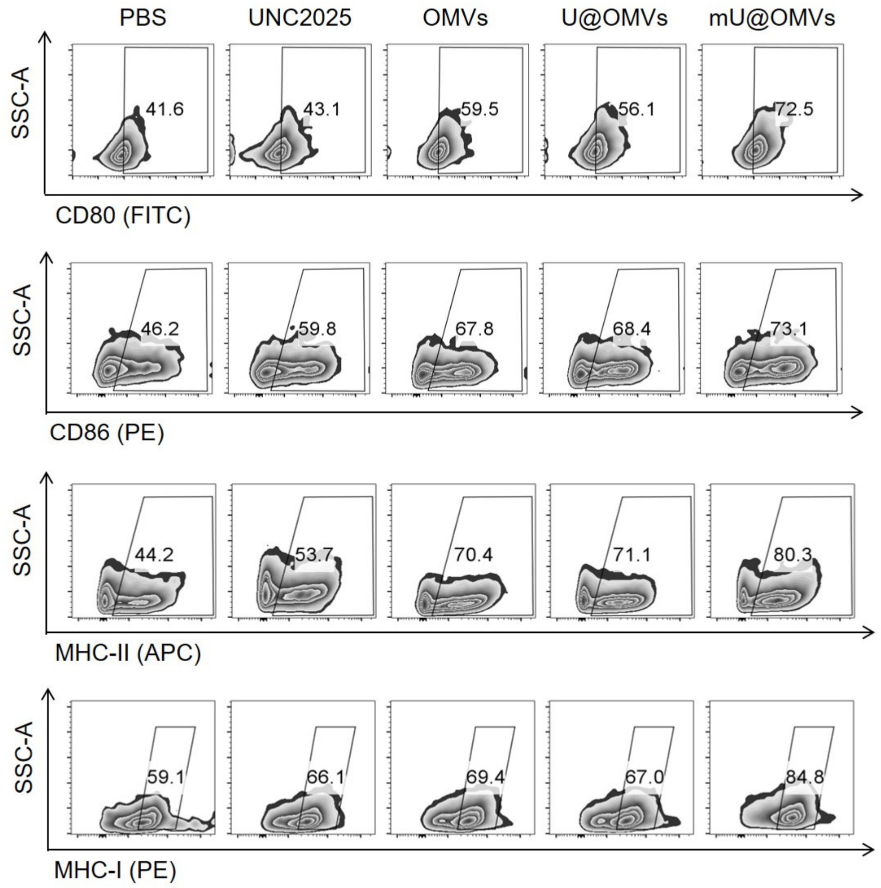

Supplementary Figure 20 Representative flow cytometry plots of CD80⁺, CD86⁺, MHC-II⁺ or MHC-I⁺ cells (gated on CD11c⁺ cells) from different treatment groups in DLNs.

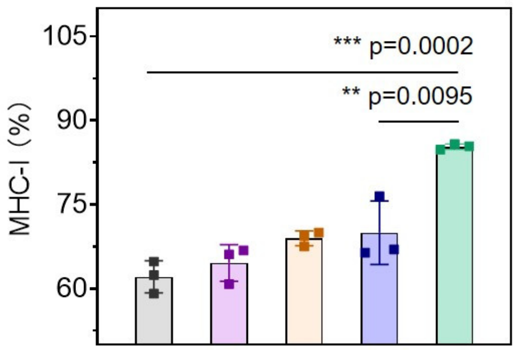

Supplementary Figure 21 Expression levels of MHC-I gated on CD11c$^+$ cells in DLNs by flow cytometry. Data are presented as mean ± s.d. (n = 3 biologically independent samples). Statistically significant differences between groups were identified by unpaired two-tailed Student's t-test. ****P < 0.0001, ***P < 0.001, **P < 0.01, *P < 0.05, n.s., not significant. Source data are provided as a Source Data file.

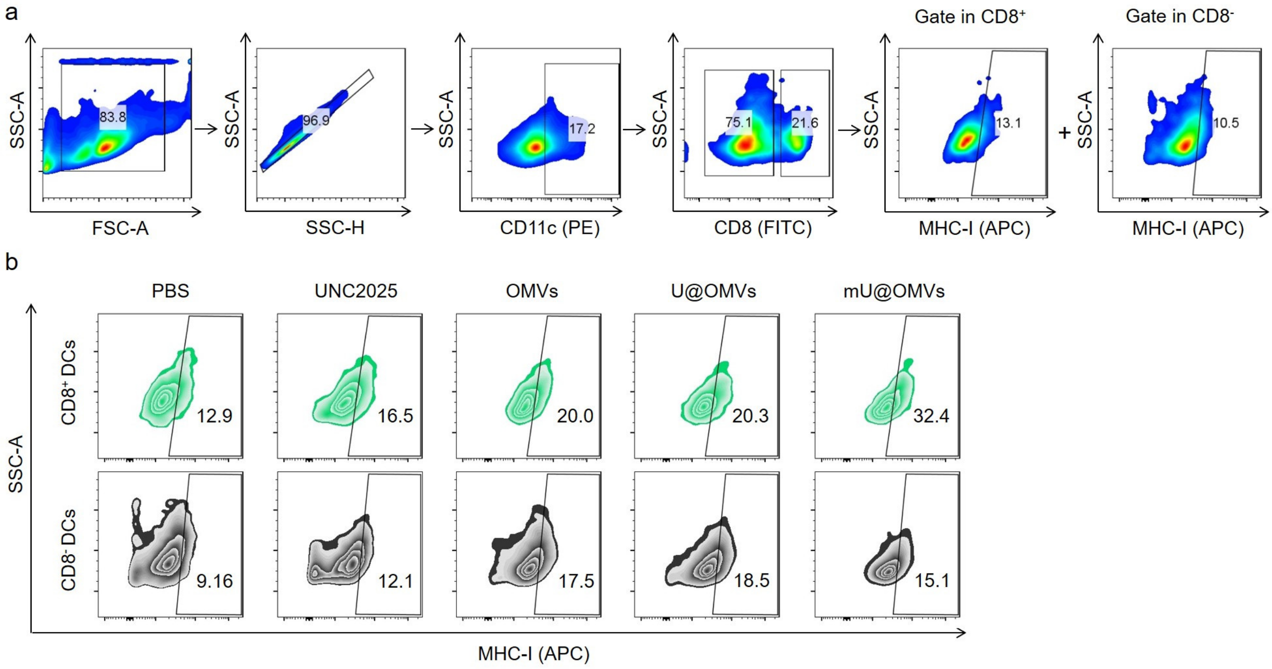

Supplementary Figure 22 mU@OMVs-mediated antigen cross-presentation by MHC class I molecules. **a** Representative flow cytometry plots of MHC-I$^+$ DCs (gated on CD11c$^+$ CD8$^+$ and CD11c$^+$ CD8$^-$ DCs) from different treatment groups in DLNs. **b** Percentages of MHC-I$^+$ DCs (gated on CD11c$^+$ CD8$^+$ and CD11c$^+$ CD8$^-$ DCs) in DLNs.

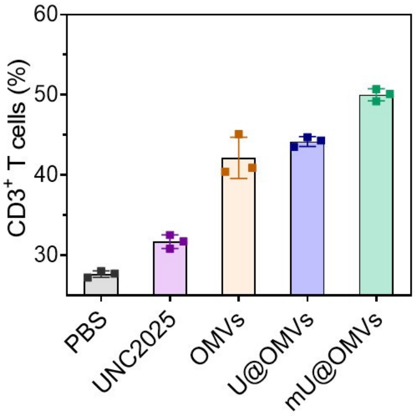

Supplementary Figure 23 The percentages of $CD3^+$ T cells from different treatment groups in spleens. Data are presented as mean ± s.d. (n = 3 biologically independent samples). Source data are provided as a Source Data file.

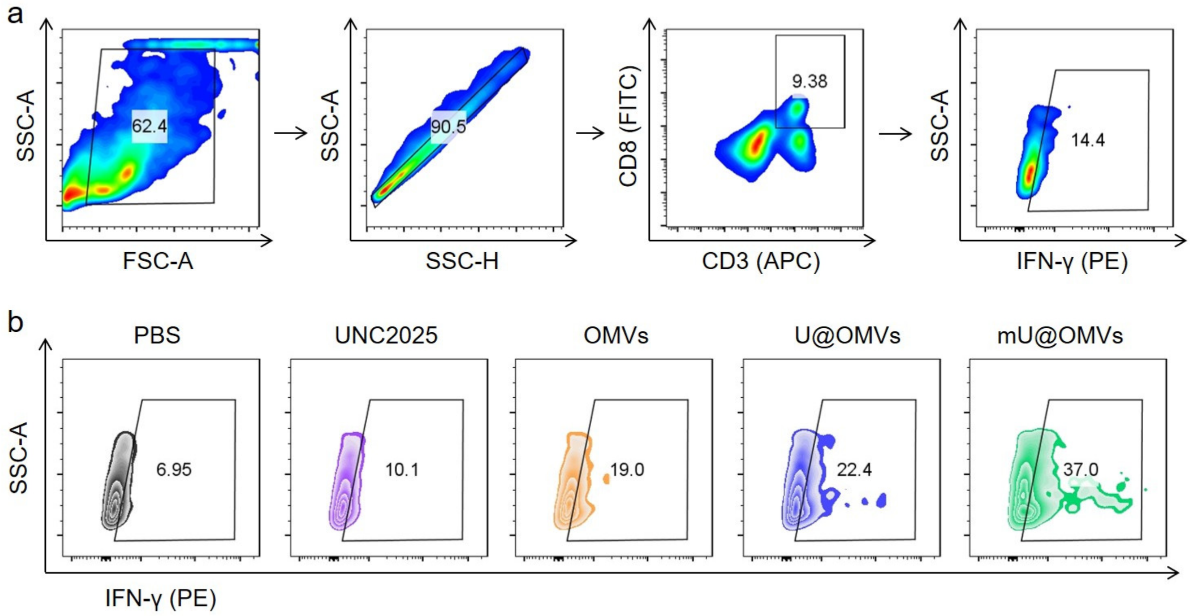

Supplementary Figure 24 Antigen-specific immune response in splenocytes after re-stimulation with the B16F10 antigen. **a** Gating strategy for flow cytometric studies of IFN-γ$^+$ T cells (gated on CD3$^+$ CD8$^+$ T cells) in splenocytes after re-stimulation with B16F10 antigen for 24 h. **b** The expression levels of IFN-γ in splenocytes (gated on CD3$^+$ CD8$^+$ T cells) after re-stimulation with B16F10 antigen for 24 h.

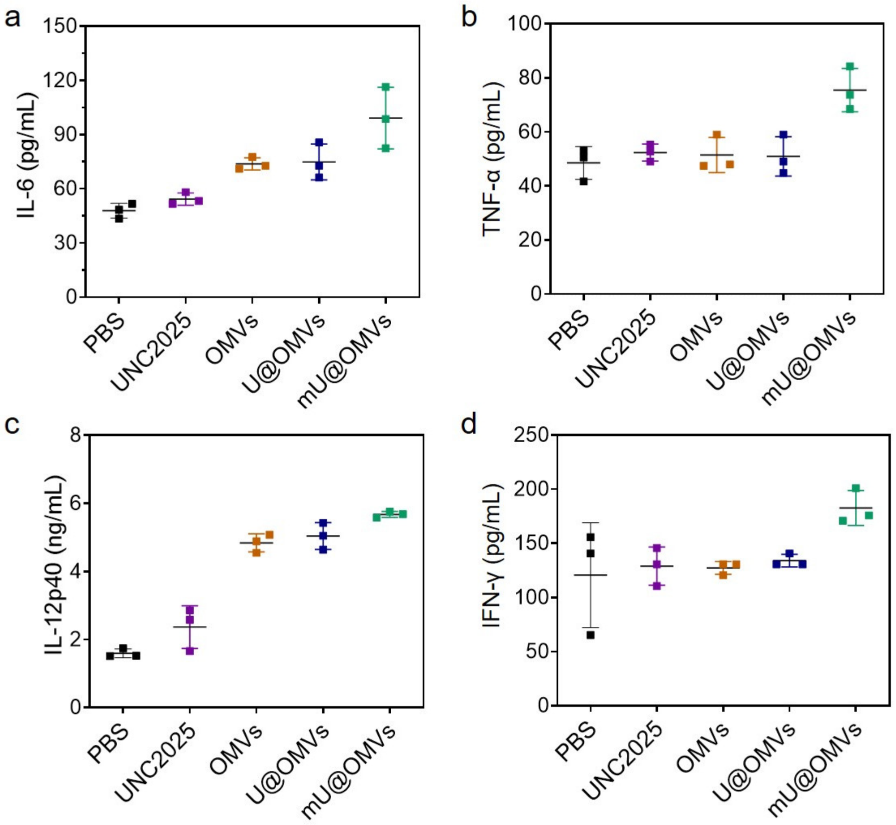

Supplementary Figure 25 The serum levels of proinflammatory cytokines, including IL-6 (**a**), TNF-α (**b**), IL-12p40 (**c**) and IFN-γ (**d**) from different groups after 48 h administration. Data are presented as mean ± s.d. (n = 3 biologically independent samples). Source data are provided as a Source Data file.

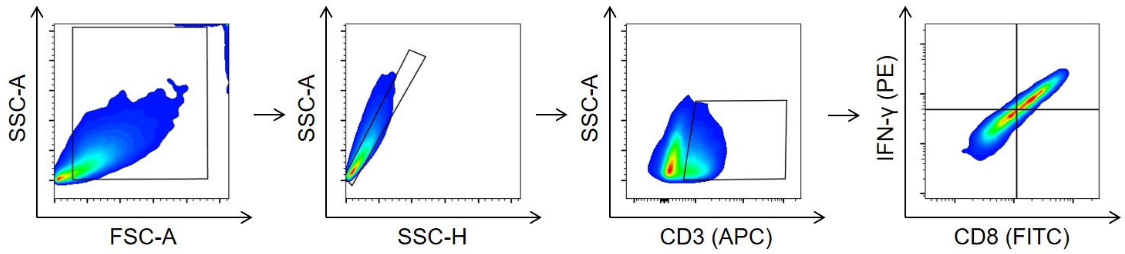

Supplementary Figure 26 Gating strategy for flow cytometric studies of IFN-γ⁺ CD8⁺ T cells (gated on CD3⁺ T cells) in tumors.

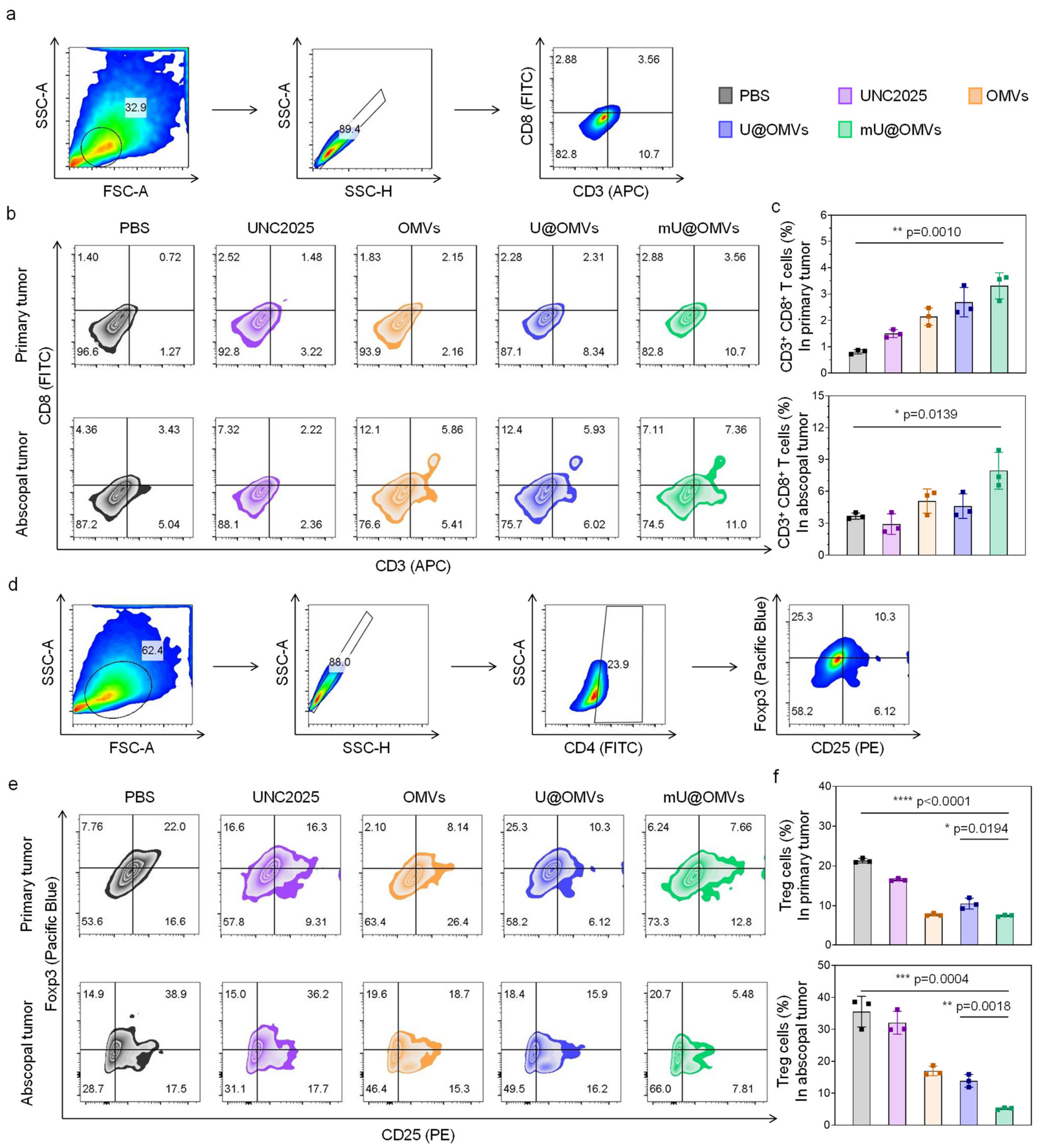

Supplementary Figure 27 T-cell infiltration in the tumor microenvironment. The tumors were isolated and analyzed on the 7th day after the final administration in a bilateral tumor model. **a** Gating strategy for flow cytometric studies of $CD3^+$ $CD8^+$ T cells in primary and abscopal tumors. **b** Flow cytometry data of $CD3^+$ $CD8^+$ T cells in primary and abscopal tumors. **c** The percentages of $CD3^+$ $CD8^+$ T cells in primary and abscopal tumors. **d** Gating strategy for flow cytometric studies of $CD4^+$ $CD25^+$ $Foxp3^+$ Treg cells in primary and abscopal tumors. **e** Flow cytometry data of Treg cells in primary and abscopal tumors. **f** The percentages of Treg cells in primary and abscopal tumors. Data are presented as mean ± s.d. (n = 3 biologically independent samples).

Statistically significant differences between groups were identified by unpaired two-tailed Student's t-test. ****P < 0.0001, ***P < 0.001, **P < 0.01, *P < 0.05, n.s., not significant. Source data are provided as a Source Data file.

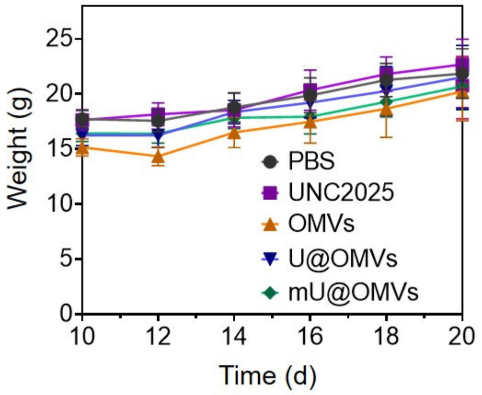

Supplementary Figure 28 Body weight during antitumor experiment against B16F10. Data are presented as mean ± s.d. (n = 9 biologically independent mice). Source data are provided as a Source Data file.

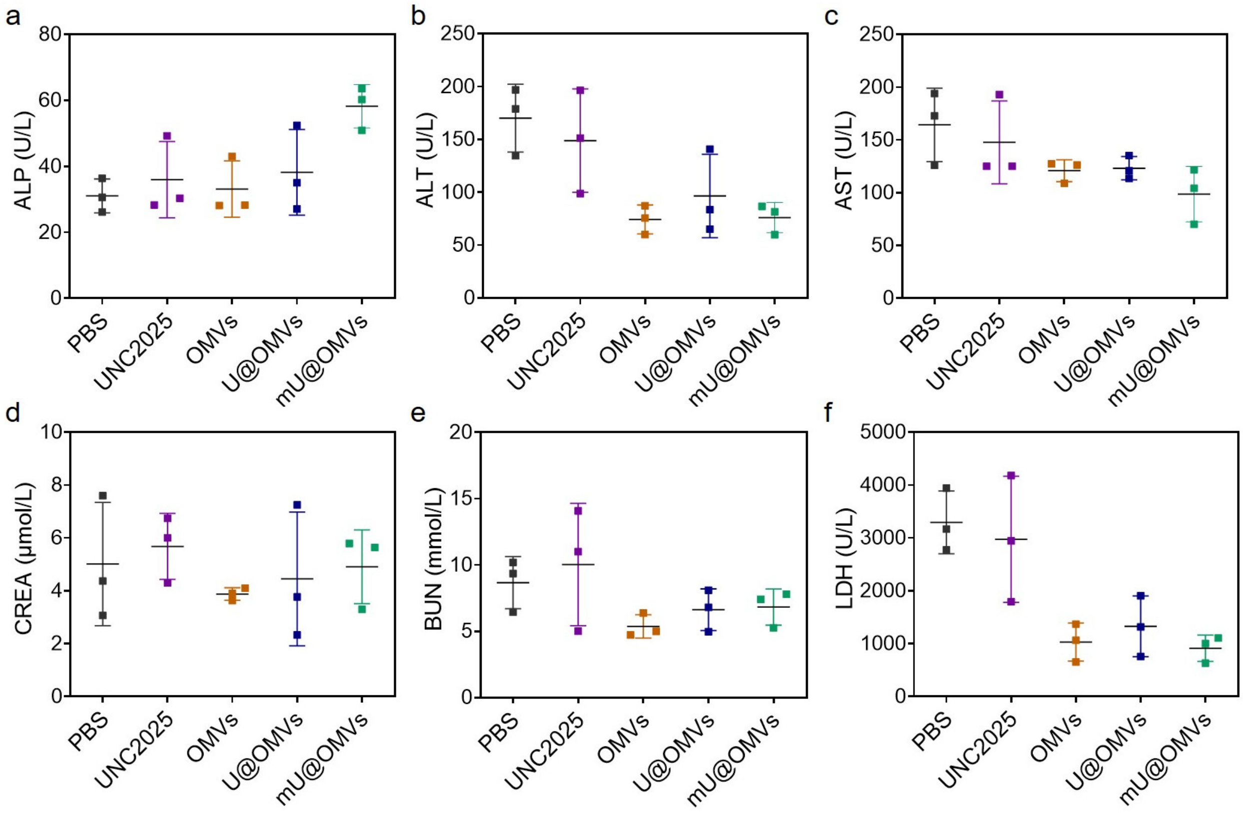

Supplementary Figure 29 Serum biochemical indexes of ALP (**a**), ALT (**b**), AST (**c**), CREA (**d**), BUN (**e**) and LDH (**f**) in different groups at the endpoint of the antitumor experiment against B16F10. Data are presented as mean ± s.d. (n = 3 biologically independent samples). Source data are provided as a Source Data file.

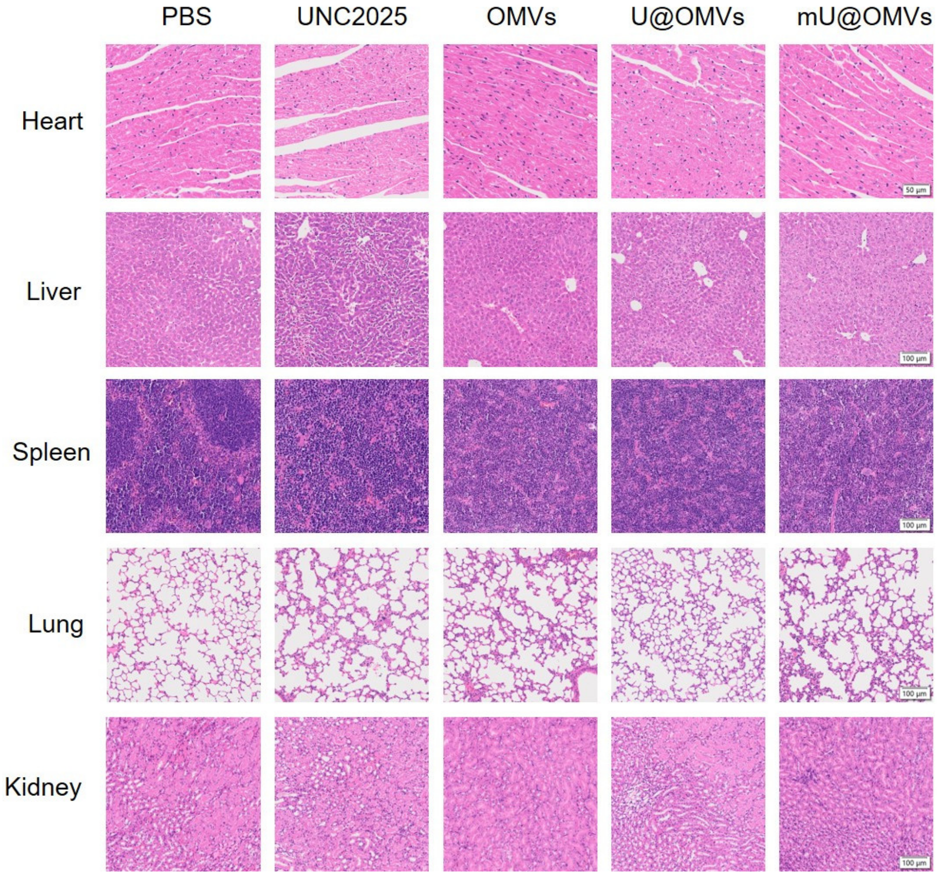

Supplementary Figure 30 H&E staining of major organs at the endpoint of the antitumor experiment against B16F10. The experiments were repeated three times independently.

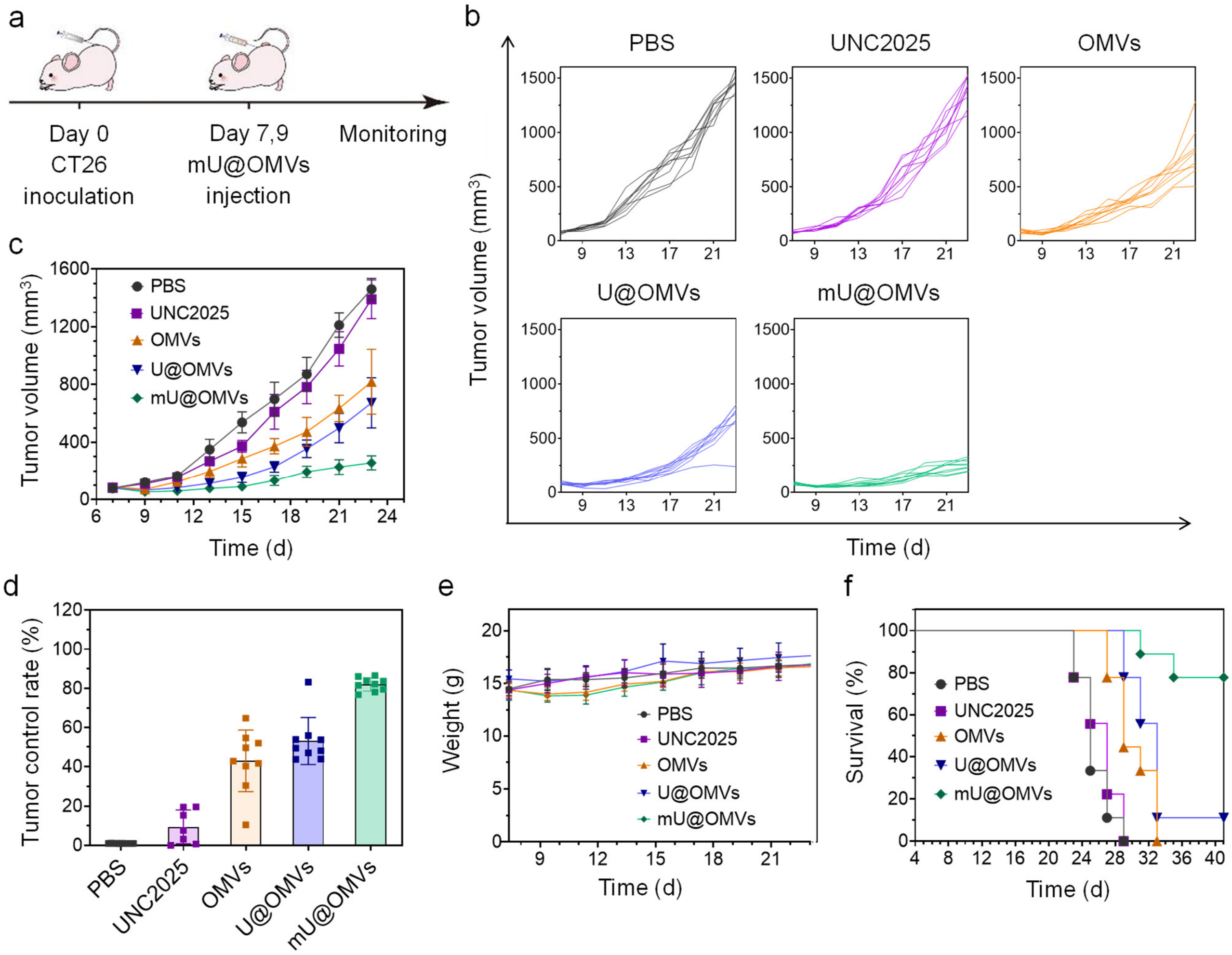

Supplementary Figure 31 The antitumor effect of mU@OMVs to inhibit CT26 tumors. **a** Schematic illustration. **b** Individual tumor growth kinetics were recorded every three days. **c** Tumor volumes. **d** Tumor control rate. **e** Body weight during antitumor experiment. **f** Survival curve of each group. Data are presented as mean ± s.d. (n = 9 biologically independent mice). Source data are provided as a Source Data file.

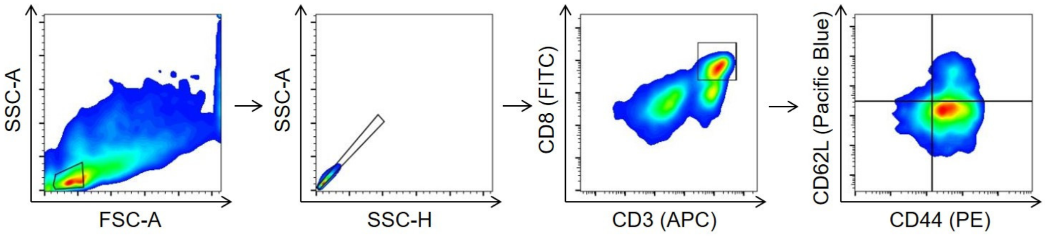

Supplementary Figure 32 Gating strategy for flow cytometric studies of $CD3^+$ $CD8^+$ T lymphocytes and $CD3^+$ $CD8^+$ $CD44^+$ $CD62L^-$ T lymphocytes (T_{EM}) in spleens.

Supplementary Table 1 The concentration (particles/mL) of OMVs, U@OMVs or mU@OMVs determined by NTA.

	OMVs	U@OMVs	mU@OMVs
Concentration (Particles/mL)	5.9×10^{8}	5.3×10^{8}	5.5×10^{8}

Supplementary Table 2 The relative abundance of proteins captured by mU@OMV.

	proteins	Intensity	Relative abundance		proteins	Intensity	Relative abundance
1	C3	680470000	10	23	Hist1h1c	76945000	1.13076256
2	Pdcd11	473620000	6.96018928	24	Hspa1a	73670000	1.08263406
3	Camk2d	415430000	6.10504504	25	Hmgb1	56022900	0.82329713
4	Eef1a1	323270000	4.75068703	26	Hsp90b1	27793000	0.40843829
5	Actg1	284760000	4.18475465	27	Hspa4	9282000	0.13640572
6	Acbd3	237220000	3.48611989	28	Brd2	455920000	6.70007495
7	Mcm2	160920000	2.36483607	29	Sept7	313240000	4.6032889
8	Flna	155970000	2.29209223	30	Smarcd1	99552000	1.46298882
9	Msn	125410000	1.84299087	31	Trip12	53341000	0.78388467
10	Plaa	99984000	1.46933737	32	Cd34	19807000	0.29107823
11	Dpysl2	79022000	1.16128558	33	Fn1	17335000	0.25475039
12	Larp1	71600000	1.05221391	34	Snx5	10005000	0.14703073
13	Gapvd1	68265000	1.00320367	35	Tubb3	367230000	5.3967111
14	Ehd2	58500000	0.85969991	36	Eef2	268740000	3.94932914
15	Dffb	58415000	0.85845078	37	Actn4	42731000	0.62796303
16	Lrp1	29595000	0.43491998	38	Smarcd1	20781000	0.30539186
17	Wdr1	22698000	0.33356357	39	Aldh18a1	15015000	0.22065631
18	Ctnnd1	12596000	0.18510735	40	Cad	12713000	0.18682675
19	Hspa8	386240000	5.67607683	41	Zc3h14	14202000	0.20870869
20	Hsp90ab1	288320000	4.23707144	42	Ddx27	6022900	0.08851088
21	H2afz	116270000	1.70867195	43	Dag1	2573900	0.03782533
22	Hsp90aa1	107740000	1.58331741				
