# Supplementary material for: Bacterial outer membrane vesicle based versatile nanosystem boosts the efferocytosis blockade triggered tumor-specific immunity
Source: Nat Commun. 2023 Mar 25;14:1675. doi: 10.1038/s41467-023-37369-0 (PMC10039929; doi:10.1038/s41467-023-37369-0)
Supplement: Supplementary file 3 — Reporting Summary [file 41467_2023_37369_MOESM3_ESM.pdf]

nature portfolio

Corresponding author(s): Hai-Yan Xie

Last updated by author(s): Feb 23, 2023

Reporting Summary

Nature Portfolio wishes to improve the reproducibility of the work that we publish. This form provides structure for consistency and transparency in reporting. For further information on Nature Portfolio policies, see our Editorial Policies and the Editorial Policy Checklist.

Statistics

For all statistical analyses, confirm that the following items are present in the figure legend, table legend, main text, or Methods section.

n/a	Confirmed	
☐	☒	The exact sample size (*n*) for each experimental group/condition, given as a discrete number and unit of measurement
☐	☒	A statement on whether measurements were taken from distinct samples or whether the same sample was measured repeatedly
☐	☒	The statistical test(s) used AND whether they are one- or two-sided<br>*Only common tests should be described solely by name; describe more complex techniques in the Methods section.*
☒	☐	A description of all covariates tested
☒	☐	A description of any assumptions or corrections, such as tests of normality and adjustment for multiple comparisons
☐	☒	A full description of the statistical parameters including central tendency (e.g. means) or other basic estimates (e.g. regression coefficient) AND variation (e.g. standard deviation) or associated estimates of uncertainty (e.g. confidence intervals)
☐	☒	For null hypothesis testing, the test statistic (e.g. *F*, *t*, *r*) with confidence intervals, effect sizes, degrees of freedom and *P* value noted<br>*Give P values as exact values whenever suitable.*
☒	☐	For Bayesian analysis, information on the choice of priors and Markov chain Monte Carlo settings
☒	☐	For hierarchical and complex designs, identification of the appropriate level for tests and full reporting of outcomes
☒	☐	Estimates of effect sizes (e.g. Cohen's *d*, Pearson's *r*), indicating how they were calculated

Our web collection on statistics for biologists contains articles on many of the points above.

Software and code

Policy information about availability of computer code

Data collection	ZetaView (Particle Metrix) was used to collect size distribution data. Eclipse-Ti2 (NiKon) was used to collest Confocal laser scanning microscope images, VS200 (Olympus) was uased to collect tissue section images. ChemiDoc XRS+ System(BIO RAD, version number: 721BR0517) was used to collect western blot images and Living Image software (PerkinElmer's IVIS® optical imaging systems) was used for the bioluminescence assay. Bioscience FACSAria (BD) was used for flow cytometry.
Data analysis	Image Lab (version number, 3.0) was used to analyze the data of western blot. FlowJo (version number, 10.0.0.0) was used to analyze the data of flow cytometry. GraphPad Prism (version number, 8.0.2.263) was used for the statistical analysis. Living Image software (version number, 4.3.1.16427) was used to analyze the data of in vivo bioluminescence assay.

For manuscripts utilizing custom algorithms or software that are central to the research but not yet described in published literature, software must be made available to editors and reviewers. We strongly encourage code deposition in a community repository (e.g. GitHub). See the Nature Portfolio guidelines for submitting code & software for further information.

Data

Policy information about availability of data

All manuscripts must include a data availability statement. This statement should provide the following information, where applicable:

- Accession codes, unique identifiers, or web links for publicly available datasets
- A description of any restrictions on data availability
- For clinical datasets or third party data, please ensure that the statement adheres to our policy

All the other data supporting the findings of this study are available within the article and its Supplementary Information file and from the corresponding author upon reasonable request.

Human research participants

Policy information about studies involving human research participants and Sex and Gender in Research.

Reporting on sex and gender	no human research
Population characteristics	no human research
Recruitment	no human research
Ethics oversight	no human research

Note that full information on the approval of the study protocol must also be provided in the manuscript.

Field-specific reporting

Please select the one below that is the best fit for your research. If you are not sure, read the appropriate sections before making your selection.

☒ Life sciences ☐ Behavioural & social sciences ☐ Ecological, evolutionary & environmental sciences

For a reference copy of the document with all sections, see nature.com/documents/nr-reporting-summary-flat.pdf

Life sciences study design

All studies must disclose on these points even when the disclosure is negative.

Sample size	No statistical methods were used to determine sample sizes. Sample sizes were primarily determined by the availability of data or on the basis of previous experimental experience (Adv. Mater., 2021, 33, e2005562; Angew. Chem. Int. Ed., 2020, 59, 406). In vitro experiments were performed with at least 3 biologically independent samples. All in vivo experiments were performed with at least 3 independent animals. Sample sizes were sufficient to perform statistical analyses.
Data exclusions	No samples were excluded.
Replication	All attempts at replication were successful. Experimental repeat numbers are provided in the figure captions.
Randomization	All samples and organisms were numbered and randomly grouped via the random-number-table method.
Blinding	All experimental procedures and the quantification of results, including injections, isolation of the tumors or organs, tissue histological analysis and flow cytometry, were done by independent researchers. All the investigators were blinded to group allocation.

Reporting for specific materials, systems and methods

We require information from authors about some types of materials, experimental systems and methods used in many studies. Here, indicate whether each material, system or method listed is relevant to your study. If you are not sure if a list item applies to your research, read the appropriate section before selecting a response.

Materials & experimental systems

n/a	Involved in the study	
☐	☒	Antibodies
☐	☒	Eukaryotic cell lines
☒	☐	Palaeontology and archaeology
☐	☒	Animals and other organisms
☒	☐	Clinical data
☒	☐	Dual use research of concern

Methods

n/a	Involved in the study	
☒	☐	ChIP-seq
☐	☒	Flow cytometry
☒	☐	MRI-based neuroimaging

Antibodies

Antibodies used

FITC-conjugated anti-E. coli LPS antibody (bs-8000R-FITC, polyclonal antibody, 1:1000 for OMVs labeling), anti-phospho-MerTK antibody (bs-18791R, polyclonal antibody, 1:2000 for western blot), anti-phospho-Axl antibody (bs-5181R, polyclonal antibody, 1:1000 for western blot) and anti-Axl antibody (bs-5180R, polyclonal antibody, 1:1000 for western blot) were purchased from Bioss (China). Anti-MerTK (ab270448, clone name: EPR23248-259, 1:1000 for western blot), anti-beta Actin (ab8227, polyclonal antibody, 1:10000 for western blot) and Goat-Anti-Rabbit IgG (HRP) antibodies (ab6721, polyclonal antibody, 1:10000 for western blot) were purchased from Abcam (UK). FITC-conjugated anti-mouse CD11c antibody (N418, clone name: AB_2883792, 1:1000 for immunofluorescence) was purchased from Proteintech (China). APC anti-mouse CD11b (101211, clone name: AB_312794, 1:500 for immunofluorescence), FITC-conjugated anti-mouse CD80 (104705, clone name: AB_313126, 1:100 for flow cytometry), PE-conjugated anti-mouse CD86 (159203, clone name: AB_2832567, 1:100 for flow cytometry), APC-conjugated anti-mouse MHC-II (107613, clone name: AB_313328, 1:100 for flow cytometry), PE-conjugated anti-mouse MHC-I (114608, clone name: AB_313599, 1:100 for flow cytometry), PE-conjugated anti-mouse CD69 (104507, clone name: AB_313110, 1:100 for flow cytometry), APC-conjugated anti-mouse CD3 (100312, clone name: AB_312677, 1:100 for flow cytometry), FITC-conjugated anti-mouse CD8 (100705, clone name: AB_312744, 1:100 for flow cytometry), FITC-conjugated anti-mouse CD4 (100405, clone name: AB_312690, 1:100 for flow cytometry), PE-conjugated anti-mouse CD25 (101903, clone name: AB_312846, 1:100 for flow cytometry), Pacific Blue-conjugated anti-mouse Foxp3 antibody (126409, clone name: AB_2247064, 1:100 for flow cytometry), PE-conjugated anti-mouse IFN-γ (505807, clone name: AB_315401, 1:100 for flow cytometry), PE-conjugated anti-mouse CD44 (103007, clone name: AB_312958, 1:100 for flow cytometry), Pacific Blue-conjugated anti-mouse CD62L antibodies (104424, clone name: AB_493380, 1:100 for flow cytometry) were purchased from BioLegend (USA). PE-conjugated anti-mouse CD206 (12-2069-42, clone name: AB_10804655, 1:100 for flow cytometry) antibody was purchased from eBioscience (USA).

Validation

All primary antibodies were purchased from the supplier, and used without additional validation. The validation of all the antibodies can be found on the manufacturers' websites:
1.FITC-conjugated anti-E. coli LPS antibody: http://www.bioss.com.cn/prolook_03_biaoji.asp?pro2a=2017122694325&pro33=206
2.anti-phospho-MerTK antibody: http://www.bioss.com.cn/prolook_03.asp?id=AF08169606022130&pro37=1
3. anti-phospho-Axl antibody: http://www.bioss.com.cn/prolook_03.asp?id=AF08169606008730&pro37=1
4. anti-Axl antibody: http://www.bioss.com.cn/prolook_03.asp?id=AF08169606008729&pro37=1
5. Anti-MerTK: https://www.abcam.cn/mertk-antibody-epr23248-259-ab270448.html
6. anti-beta Actin: https://www.abcam.cn/beta-actin-antibody-ab8227.html
7. Goat-Anti-Rabbit IgG (HRP) antibodies: https://www.abcam.cn/goat-rabbit-igg-hl-hrp-ab6721.html
8. FITC-conjugated anti-mouse CD11c antibody: https://www.ptgcn.com/products/CD11c-Antibody-FITC-65130.htm
9. APC anti-mouse CD11b: https://www.biolegend.com/ja-jp/products/apc-anti-mouse-human-cd11b-antibody-345
10. FITC-conjugated anti-mouse CD80: https://www.biolegend.com/ja-jp/products/fitc-anti-mouse-cd80-antibody-41
11. PE-conjugated anti-mouse CD86: https://www.biolegend.com/ja-jp/products/pe-anti-mouse-cd86-antibody-18945
12. APC-conjugated anti-mouse MHC-II: https://www.biolegend.com/ja-jp/products/apc-anti-mouse-i-a-i-e-antibody-2488
13. PE-conjugated anti-mouse MHC-I: https://www.biolegend.com/ja-jp/products/pe-anti-mouse-h-2k-b-h-2d-b-antibody-1686
14. PE-conjugated anti-mouse CD69: https://www.biolegend.com/ja-jp/products/pe-anti-mouse-cd69-antibody-265
15. APC-conjugated anti-mouse CD3: https://www.biolegend.com/ja-jp/products/apc-anti-mouse-cd3epsilon-antibody-21
16. FITC-conjugated anti-mouse CD8: https://www.biolegend.com/ja-jp/products/fitc-anti-mouse-cd8a-antibody-153
17. FITC-conjugated anti-mouse CD4: https://www.biolegend.com/ja-jp/products/fitc-anti-mouse-cd4-antibody-248
18. PE-conjugated anti-mouse CD25: https://www.biolegend.com/ja-jp/products/pe-anti-mouse-cd25-antibody-129
19. Pacific Blue-conjugated anti-mouse Foxp3 antibody: https://www.biolegend.com/ja-jp/products/pacific-blue-anti-mouse-foxp3-antibody-4663
20. PE-conjugated anti-mouse IFN-γ: https://www.biolegend.com/ja-jp/products/pe-anti-mouse-ifn-gamma-antibody-997
21. PE-conjugated anti-mouse CD44: https://www.biolegend.com/ja-jp/products/pe-anti-mouse-human-cd44-antibody-2206
22. Pacific Blue-conjugated anti-mouse CD62L antibodies: https://www.biolegend.com/ja-jp/products/pacific-blue-anti-mouse-cd62l-antibody-3117
23. PE-conjugated anti-mouse CD206: https://www.thermofisher.cn/cn/zh/antibody/product/CD206-MMR-Antibody-clone-19-2-Monoclonal/12-2069-42

Eukaryotic cell lines

Policy information about cell lines and Sex and Gender in Research

Cell line source(s)

CT26 (mouse colon cancer cell line) and B16F10 (murine melanoma cancer cell line) were kindly obtained from the Institute of Process Engineering (China). RAW264.7 (murine macrophage cell line) was purchased from Peking Union Medical College Hospital (catalog number: 1101MOU-PUMC000146).

Authentication

We did not authenticate CT26, B16F10 and RAW264.7 cells.

Mycoplasma contamination	The CT26, B16F10 and RAW264.7 cell lines tested negative for mycoplasma contamination.
Commonly misidentified lines (See ICLAC register)	No commonly misidentified cell lines were used.

Animals and other research organisms

Policy information about studies involving animals; ARRIVE guidelines recommended for reporting animal research, and Sex and Gender in Research

Laboratory animals	Female BALB/c mice and C57BL/6 mice were purchased from Beijing Vital River Laboratory Animal Technology Co. Ltd (Beijing, China). Mice used in these studies were 6 weeks old in the start of the experiment. Mice were housed in a room with a temperature of 20-22 °C and and a humidity of 30–70%.
Wild animals	The study did not involve wild animals.
Reporting on sex	This research has no sex-based analysis.
Field-collected samples	The study did not involve samples collected from the field.
Ethics oversight	All animal experiments were performed under the guidance of the Beijing Animal Ethics Association and the Ethics Committee of Beijing Institute of Technology (approval ID: 2019-0010-M-2020019).

Note that full information on the approval of the study protocol must also be provided in the manuscript.

Flow Cytometry

Plots

Confirm that:

☒ The axis labels state the marker and fluorochrome used (e.g. CD4-FITC).

☒ The axis scales are clearly visible. Include numbers along axes only for bottom left plot of group (a 'group' is an analysis of identical markers).

☒ All plots are contour plots with outliers or pseudocolor plots.

☒ A numerical value for number of cells or percentage (with statistics) is provided.

Methodology

Sample preparation	Murine bone marrow-derived dendritic cells (BMDCs) was prepared by the following steps:<br>1. Under sterile conditions, isolate bone marrow femurs from mice, and place into a sterile cell culture dish.<br>2. Use forceps and small scissors to cut away the muscle and fibrous tissues from the bone. If muscles are still intact, wipe the bones with a saturated with 70 % ethanol to remove any excess muscle fibers.<br>3. Cells were collected into PBS and then ground through a cell screen with a pore size of 40 μm.<br>4. The obtained cells were centrifugated at 800 × g and room temperature for 5 min.<br>5. Discarded the supernatant, added the red cell lysate (2 mL/spleen) to the pellet and incubated for 8 min.<br>6. Samples were centrifuged at 800 × g for 5 min and resuspended pellets to a new sterile cell culture dish with RPMI 1640 medium with fetal bovine serum to a final concentration of 10 %.<br>7. Cells from marrow cavities of femurs and tibias of C57BL/6 mice were cultivated in plates with a medium containing 20 ng/mL GM-CSF and 20 ng/mL IL-4 for 7 days.<br><br>The spleen cell suspension was prepared by the following steps:<br>1. Spleens were collected into medium containing FBS and then ground through a cell screen with a pore size of 40 μm.<br>2. The obtained splenocytes were centrifugated at 800 × g and room temperature for 5 min.<br>3. Discarded the supernatant, added the red cell lysate (2 mL/spleen) to the pellet and incubated for 8 min.<br>4. Samples were centrifuged at 800 × g for 5 min and resuspended pellets in medium.
Instrument	FACSAria (BD) was used to analyze samples.
Software	FlowJo (version number, 10.0.0.0) was used to analyze the data of flow cytometry.
Cell population abundance	The cell population abundance was detected by flow cytometer and described in the manuscript.
Gating strategy	In general, cells were first gated on FSC/SSC. Singlet cells were gated using SSC-H and SSC-A. The cell populations within the gate were further analyzed based on expression of markers. The detailed gating strategy could be found in supplementary information.

☒ Tick this box to confirm that a figure exemplifying the gating strategy is provided in the Supplementary Information.
